# Effect of addition of titanium dioxide nanoparticles on the antimicrobial properties, surface roughness and surface hardness of polymethyl methacrylate: A Systematic Review.

**DOI:** 10.12688/f1000research.130028.1

**Published:** 2023-05-31

**Authors:** Pragati Kaurani, Amit D Hindocha, Rasika Manori Jayasinghe, Umesh Y Pai, Kavita Batra, Carrie Price

**Affiliations:** 1 Department of Prosthodontics, Mahatma Gandhi Dental College and Hospital, Jaipur, Rajasthan, 302022, India; 2 Department of Prosthodontics, Sinhgad Dental College and Hospital, Pune, Maharashtra, 411041, India; 3 Department of Prosthetic Dentistry, Faculty of Dental Sciences , University of Peradeniya, Kandy, Central province, 20400, Sri Lanka; 4 Department of Prosthodontics, Manipal College of Dental Sciences, Mangalore, Karnataka, 575004, India; 5 Department of Medical Education, Kirk Kerkorian School of Medicine at University of Nevada, Las Vegas, Nevada, 89102, USA; 6 Health Professions Librarian, Albert S. Cook Library, Towson University, Towson, Maryland, 21252, USA

**Keywords:** Nanoparticles, titanium dioxide nanoparticles, mechanical properties, antimicrobial properties, denture base resins

## Abstract

**Background: **Polymethyl Methacrylate (PMMA) denture-base resins have poor surface properties that facilitates microbial adhesion causing denture stomatitis. This systematic review aims to evaluate the effect of different sizes and percentages of titanium dioxide nanoparticles (TiO2NP) on the antimicrobial property, surface roughness and surface hardness of PMMA denture base resin.

**Methods:** A systematic search of English peer-reviewed articles, clinical trial registries, grey literature databases and other online sources was performed using the PRISMA-S Guidelines for *In-Vivo* and *In-Vitro* studies. Qualitative data synthesis was performed to analyse sample dimensions, acrylic used, treatments of nanoparticles, methods used for testing and effect of size and percentage of nanoparticle. Risk of bias assessment was done using modified Cochrane risk of bias tool.

**Results: **Out of 1376 articles, 15 were included. TiO _2_NP of size less than 30 nm was most frequently used. Both antimicrobial property and surface hardness improved irrespective of the size of the added TiO _2_NP. Three studies reported increase in the surface roughness with less than 50 nm TiO _2_NP.  3% TiO _2_NP was most frequently used. On increasing the percentage, three studies reported an increase in antimicrobial property, while two studies found no change. With TiO _2_NP greater than or equal to 3%, six studies reported an increase in surface hardness, while two reported increase in surface roughness. Large methodological variations were observed across studies. All studies except one were of moderate quality.

**Conclusions:**   On addition of TiO _2_NP to heat polymerized PMMA, the antimicrobial property and surface hardness improved irrespective of the size of the TiO _2_NP, however, addition of nanoparticles less than 50 nm increased the surface roughness. Increasing the percentage of TiO _2_NP increased the surface hardness but did not always increase the antimicrobial property. Addition of 3% TiO _2_NP provided optimum results with regards to antimicrobial effect and surface hardness, but increase in the surface roughness.

## Introduction

Polymethyl methacrylate (PMMA) is a widely used denture base material and its extensive usage can be attributed to certain advantages, such as ease in manipulation, achieving good finish and polish, not requiring expensive processing equipment, stability in the dynamic oral environment and biocompatibility with the oral tissues. ^1^^,^^2^ Despite these advantages, it has surface properties like roughness, surface porosities, free surface energy and contact angle that facilitate adhesion of microorganisms, such as Candida Albicans. ^3^^–^^5^ These microbial adhesions lead to the formation of denture biofilm, which eventually leads to denture stomatitis, an oral condition with reportedly high incidence that affects nearly 65% of denture wearers. ^6^^,^^7^ Therefore, numerous efforts are being made to improve the mechanical and biological properties in PMMA by making modifications in the structure or by nanoscale reinforcing additives.

The recently introduced advances in nanotechnology aim at improving antimicrobial and mechanical properties of dental materials by the addition of nanoparticles (NPs). ^8^^–^^10^ The mixture of polymer matrix and fillers at the nanoscale makes a new polymeric nanocomposite. ^11^ Studies have shown that addition of such nanoscale reinforcing additives lead to the formation of a nanocomposite, which shows improved mechanical and biological properties. ^12^ The properties of the resulting new nanocomposite depend on several factors, such as size, morphology and quantity of NPs being added. ^13^

Among the various NPs that have been used, metal oxides particularly have gained more attention owing to their proven antimicrobial effects. ^14^^,^^15^ Among others, the effect of addition of titanium dioxide nanoparticles (TiO _2_NP) on the mechanical and biological properties of PMMA has also been extensively studied. ^1^^,^^2^^,^^8^ Given the advantages and properties of TiO _2_NP (e.g. non-toxicity, chemical stability, white in color, biocompatibility, availability and cost-effectiveness), makes it a highly desirable additive. ^16^^–^^18^ Moreover, TiO _2_NP has the intrinsic antimicrobial properties due to the production of cytotoxic oxygen radicals, thereby providing antimicrobial effect to PMMA denture base. ^19^ With the addition of NPs, certain properties of PMMA are observed to improve, however, when added into the polymer matrix, NPs can alter the surface roughness and surface hardness. ^18^ Such alterations on the surface properties may occur due to the distribution and concentration of the NPs within the acrylic matrix. ^20^

Given the observed effects of TiO _2_NP on the antimicrobial, surface roughness (SR) and surface hardness (SH) properties of PMMA denture base resin, numerous studies have been undertaken to analyse these effects at varying concentrations of the NPs. Further, studies are being undertaken to analyse effects of nanohybrid composites, in which two or more NPs are mixed, such as TiO _2_NP mixed with another metal oxide NP (hybrid form), with the aim of obtaining improved properties or having a synergistic effect. ^21^

For a better understanding of the effect TiO _2_NP (in pure or hybrid form) on the antimicrobial, surface roughness and surface hardness of PMMA and their possible clinical usage, there is a need to produce collective evidence utilizing a systematic and robust method. Therefore, the objective of the current systematic review was to analyse the effect of addition of different sizes and percentages of TiO _2_NP on the antimicrobial, surface roughness and surface hardness of TiO _2_NP impregnated heat polymerized PMMA denture base resin fabricated using conventional methods or Computer-Aided Design/Computer-Aided Manufacturing (CAD/CAM) when compared to heat polymerized PMMA.

## Methods

### Protocol registration

The study is reported in accordance with the PRISMA 2020 guidelines. ^22^ The study protocol was registered on PROSPERO ( https://www.crd.york.ac.uk/prospero/#aboutpage), with registration number CRD42021252315 and was revised once as agreed upon by all authors. ^23^

### Eligibility criteria

The eligibility criteria for inclusion of studies were defined using the PICOS criteria, Population (P) = PMMA heat polymerized or manufactured by CAD/CAM. Intervention (I) = Addition of TiO _2_NP in pure or hybrid form in PMMA. Studies based on TiO _2_ nanotubes, fillers, fibres and coating on PMMA were excluded. Control (C) = Heat polymerized PMMA without any addition of NPs or fibres. Outcome measure (O) = Antimicrobial properties, SR and SH of PMMA. Study designs (S) = In-Vivo and In-Vitro studies were included. Studies based on TiO _2_ nanotubes, fillers, fibres and coating on PMMA were excluded. Case-reports, systematic and narrative reviews, letters to the editor, short commentaries, pilot studies or studies with preliminary results and studies that were not related to the field of dentistry were excluded.

### Search methods for identification of studies

A comprehensive search strategy was developed for each database by an experienced librarian [C.P] and sent for peer reviewing using PRESS-Guidelines. ^24^ Electronic searches in various databases were performed and reported using the PRISMA-S search extension wherein searches were done in databases, study registries, grey literature and other online sources. ^25^ Based on the PRISMA-S guidelines, a rerun of the search was done with a gap of six months to search studies between January 2000 to April 2022, and carried out in three databases PubMed (NCBI), WHO Virtual Health Library and Cochrane Library. ^25^ Two clinical trial registries ( Clinicaltrials.gov and Clinical Trials Registry of India), six other online sources (Ingenta Connect, Google Scholar, Research Gate, Dimension, Crossref and The Lens) and three sources for grey literature (BASE, OpenGrey and Grey Literature Report) were also searched. The PubMed search included both controlled vocabulary (Medical Subject Headings, or MeSH) and keyword terms for the concepts of “nanoparticles”, “titanium dioxide”, “PMMA”, “dentures”, “CAD/CAM”, “surface roughness”, “surface hardness”, “antibacterial” and “antifungal” and were used as the basis for all searches in other sources, with no usage of published search filters. Citation chasing and manual citation searching was performed of the reference lists of all the included articles by one researcher and E-mail alerts were set. The details of all the sources and their respective searches can be found in the (Supplementary Table 1, available online (Pragati, 2022)). De-duplication was performed using EndNote software.

### Selection of studies and data extraction

Initially, two researchers (P.K. and A.H.) independently screened the titles and abstracts of the exported articles in Rayyan Software ( https://www.rayyan.ai/). Full-text articles were retrieved for potentially relevant articles and screened using the eligibility criteria by the same researchers. Wherever the full articles could not be retrieved, the corresponding authors were contacted, and the articles were obtained.

Antimicrobial Property was referred to as a collective term when there was an inhibition of the growth of bacteria and fungi, prevention of the formation of microbial colonies or destruction of microorganisms. Studies showing results of TiO _2_NP potential for the treatment of denture stomatitis were also included. Studies without a suitable control group, where the studies evaluated the antimicrobial capacity of the nanoparticle material by themselves, independently of their incorporation into PMMA were excluded. In case of disagreements, conflicts were resolved, however, articles in which the conflicts could not be resolved, the two reviewers (U.P. and M.J.) were consulted for the final decision. Two researchers (P.K. and A.H.) independently studied each of the included articles and extracted the data. (Supplementary Tables 1 and 2, available online (Pragati, 2022)) For data that was found to be missing, NS (not stated) was used and when not clear, ‘unclear’ was written.

### Methodological quality and risk of bias assessment

The assessment of the quality of the included articles was performed using the modified Consolidated Standards of Reporting Trials (CONSORT) guidelines. ^26^ The selected parameters were reported as “yes” or “no” on the screening of articles ( Table 1). The risk of bias was assessed individually by two researchers P.K. and A.H. and any disagreements were resolved by the third reviewer U.P. based on the modified Cochrane Risk of Bias tool and scored as described in previous study ^27^ ( Table 2).

**Table 1.** Methodological characteristics of the included studies based on the modified CONSORT criteria.

Sr No.	Author	1	2a	2b	3	4	5	6	7	8	9	10	11	12	13	14	Total
1	Alhotan A. ^44^	Y	Y	Y	Y	Y	N	N	N	N	N	Y	Y	N	Y	Y	9/15
2	Alrahlah A. ^45^	Y	Y	Y	Y	Y	N	N	N	N	N	Y	Y	N	Y	N	8/15
3	Alwan S.A. ^46^	Y	Y	N	Y	Y	N	N	N	N	N	Y	Y	N	N	N	6/15
4	Anehosur G.V. ^47^	Y	Y	Y	Y	Y	Y	N	N	N	N	Y	Y	Y	Y	N	10/15
5	Ashour Ahmed M. ^48^	Y	Y	Y	Y	Y	N	N	N	N	N	Y	Y	N	Y	N	8/15
6.	Azemy E. ^49^	Y	Y	Y	Y	Y	Y	N	N	N	N	Y	Y	Y	N	Y	10/15
7	Bangera M.K. ^50^	Y	Y	Y	Y	Y	Y	N	N	N	N	Y	Y	Y	Y	N	10/15
8.	Casiocone M. ^51^	Y	Y	Y	Y	Y	N	N	N	N	N	Y	Y	N	Y	N	8/15
9.	Chowdhury R. ^52^	Y	Y	Y	Y	Y	Y	N	N	N	N	Y	Y	Y	Y	N	10/15
10.	Giti R. ^53^	Y	Y	Y	Y	Y	N	N	N	N	N	Y	Y	Y	Y	Y	10/15
11.	Mansour M. M. ^54^	Y	Y	Y	Y	Y	N	N	N	N	N	Y	Y	Y	N	N	8/15
12.	Ragheb N. ^55^	Y	Y	Y	Y	Y	Y	Y	Y	Y	Y	Y	Y	Y	Y	N	14/15
13.	Rashahmadi S. ^56^	Y	Y	N	Y	Y	N	N	N	N	N	Y	Y	N	N	N	6/15
14.	Song R. ^57^	Y	Y	Y	Y	Y	N	N	N	N	N	Y	Y	Y	N	N	8/15
15.	Thabet Y. ^58^	Y	Y	Y	Y	Y	N	N	N	N	N	Y	Y	Y	N	N	8/15

1: Structured Summary of trial design, methods, results and conclusions, 2a. Scientific background and explanation of rationale, 2b. Specific objectives and/or hypothesis, 3. Intervention of each group, including how and when it was administered with sufficient detail to enable replication, 4. Completely defined, pre-specified primary and secondary measures of outcome including how and when they assessed, 5. How the sample size was determined, 6. Method used to generate random allocation sequence, 7. Mechanism used to implement the random allocation sequence, 8. Generated the random sequence, 9. Blinded after assignment to intervention, 10. Statistical methods to compare groups, 11. Results for each group and estimated size of effect and its precision, 12. Limitations of trials, addressing sources of potential bias, imprecision and if relevant multiplicity of analysis,13. Sources of funding, 14. Where full protocol can be addressed. Y=Yes, N=No.

**Table 2.** Summary of Risk of Bias assessment of included studies (adapted and modified from Cochrane Risk of Bias Tool).

Sr No	Author	Allocation of concealment	Sample size calculation	Blinding	Testing methodology	Selective outcome reporting	Overall score	Risk
1	Alhotan A. ^44^	2	1	2	0	0	5	Moderate
2	Alrahlah A. ^45^	2	2	2	0	0	6	Moderate
3	Alwan S.A. ^46^	2	2	2	0	0	6	Moderate
4	Anehosur G.V. ^47^	2	0	2	0	0	4	Moderate
5	Ashour Ahmed M ^48^	2	2	2	0	0	6	Moderate
6.	Azemy E. ^49^	2	0	2	0	0	4	Moderate
6	Bangera M.K. ^50^	2	0	2	0	0	4	Moderate
7	Casiocone M. ^51^	2	2	2	0	0	6	Moderate
8	Chowdhury R. ^52^	2	0	2	0	0	4	Moderate
9	Giti R. ^53^	2	2	2	0	0	6	Moderate
11	Mansour M.M. ^54^	2	2	2	0	0	6	Moderate
13	Ragheb N. ^55^	2	0	0	0	0	2	Low
14	Rashahmadi S. ^56^	2	2	2	0	0	6	Moderate
15	Song R. ^57^	2	2	2	0	0	6	Moderate
16	Thabet Y. ^58^	2	2	2	0	0	6	Moderate

Risk of Bias tool: modified Cochrane Risk of Bias Assessment Tool. The scoring was done as: 0=All sections were clear, 1=insufficiently/unclear, 2=unrevealed. Total score was interpreted as: 0-3 =Low risk, 4-7=Moderate Risk, 8-10=High Risk.

Due to the large methodological heterogeneity observed, narrative synthesis of the data was performed and quantitative analysis of the data was not performed.

## Results

**Figure 1.**
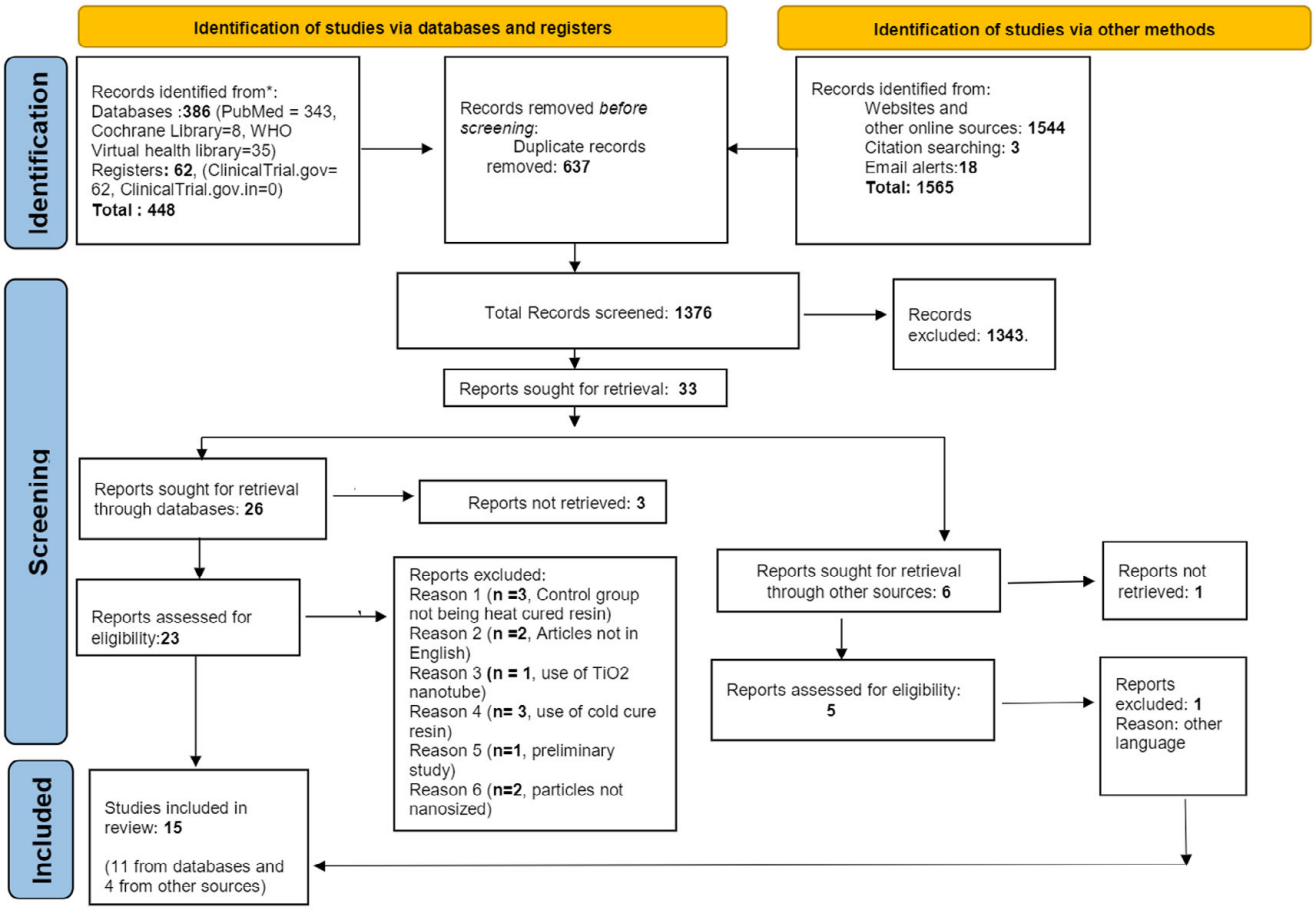
PRISMA 2020 flowchart of all the sources searched and the results obtained.

The systematic search identified 386 articles through the three databases searched, 62 articles were found from trial registries and 1565 from other sources (online sources, citation searching and email alerts). After de-duplication, 637 articles were removed and subsequently, 37 potentially relevant articles were accessed for analysis of the full text. Out of these, 17 studies were excluded due to the following reasons: articles in which TiO _2_ was added as a nanotube or coating, ^28^ studies carried out on auto-polymerized acrylic resin, ^29^^–^^31^ clinical trial reports in which full reports could not be retrieved or were not complete, ^32^^–^^35^ control group was not heat polymerized PMMA, ^36^^–^^38^ articles not in English, ^39^^–^^41^ article published as a preliminary study, ^42^ particle size was not nano. ^21^^,^^43^ The details of the articles excluded can be found in the (Supplementary Table 2, online (Pragati, 2022)). Finally, 15 articles that fulfilled the inclusion criteria were included and is depicted as modified PRISMA-2020 flow diagram made most optimal for the current review. ^44^^–^^58, 59^ ( Figure 1).

Out of 15 studies included 14 were in-vitro studies and one was a randomized control trial, 7 studies evaluated antimicrobial properties of TiO _2_NP, ^45^^,^^47^^,^^51^^,^^53^^,^^54^^,^^57^^,^^58^ 5 studies evaluated SR, ^46^^,^^50^^,^^51^^,^^52^^,^^58^ 8 studies evaluated SH ^44^^–^^46^^,^^48^^–^^50^^,^^54^^,^^56^ and 2 studies evaluated both. ^46^^,^^50^ The methodological quality assessments revealed that only one study scored above 90% ^55^ ( Table 1). The risk of bias assessment revealed moderate risk of bias for all except one study that showed a low risk of bias ^55^ ( Table 2). None of the studies used random allocation sequence, five studies provided information on sample size, ^47^^,^^49^^,^^50^^,^^52^^,^^55^ and only one study provided information on blinding. ^55^

All the studies used conventional heat polymerized PMMA with only one study that evaluated the effect of addition of TiO _2_NP on high impact heat polymerized PMMA and only one study used hybrid NPs. ^48^^,^^57^ Considerable variations in the size of TiO _2_NP used was observed, the particle sizes varied from being less than 15 nm to 100 nm. TiO _2_NP of the size less than 30 nm, was found to be used most frequently across studies. Similarly, the percentage of TiO _2_NP added varied considerably ranging from 0.1% to 7.5% with 3% by weight being the most frequently used percentage (Supplementary Tables 1 and 2, available online (Pragati, 2022)).

All studies reported an increased anti-microbial effect, irrespective of the size of addition of TiO _2_NP, but different effects was observed on increasing the percentage of the addition of TiO _2_NP. Three studies found an increase in the antimicrobial properties, while two studies did not report improved antimicrobial properties on increasing the percentage of addition of TiO _2_NP. Cascione *et al.* found a reduction in the Candida colonization area by 19% on addition of 3% TiO _2_NP compared to the 16% reduction observed with 1% TiO _2_NP. ^51^ On similar lines one study found that on increasing the percentage of TiO _2_NP the antimicrobial effect increased from 1% to 3%, while another study found that on increasing the percentage of TiO _2_NP from 1% to 3%, the bacterial adhesion of E. faecalis and P. aeruginosa reduced to 50% and 92% respectively. ^45^^,^^55^ Contrary to this, despite showing antimicrobial effect, Giti and others found no significant increase in the antimicrobial properties with increase in the percentage from 2.5% to 7.5%. ^53^ Similarly, Song and others found better results on using 0.3% of TiO _2_NP compared to 0.4% and 0.5%. ^57^

With regards to the SR, Cascione and others found a decrease in SR with large sized TiO _2_NP (greater or equal to 50 nm), ^51^ but three studies reported an increase in SR with the addition of less than 50 nm TiO _2_NP, ^46^^,^^50^^,^^52^ and one study found in-significant improvement in SR. ^58^ Three studies found an increase in the SR on addition of 3% and 2% TiO _2_NP, ^46^^,^^50^^,^^52^ while one study found 54% and 72% roughness reduction with 1% and 3% TiO _2_NP, ^51^ and one study found an insignificant increase in SR with 5% TiO _2_NP. ^58^

Contrary to the effect on SR but similar to antimicrobial effect, SH was found to increase irrespective of the size of the added TiO _2_NP. Studies that used TiO _2_NP less than 50 nm particle size ^41^^,^^46^^,^^48^^,^^54^^,^^56^^,^^50^ as well as greater than 50 nm reported improved SH. ^45^ Further, the results indicate that on addition of TiO _2_NP greater than or equal to 3%, there is an observed increase in the SH. ^44^^–^^46^^,^^48^^,^^49^^,^^54^On the other hand, when used less than 3%, TiO _2_NP either caused no change or reduction in SH. ^44^^,^^48^^,^^50^Alhotan *et al.* found no improvement in the hardness by adding 1.5% TiO _2_NP, ^44^ Ashur Ahmed and others found no significant difference on adding 1% TiO _2_NP and Bangera and others found values close to the control groups on addition of 1% TiO _2_NP. ^45^^,^^50^ The only exception to this were Alrahlah and others, who found an 18% and 24% increase in the SH on addition of 1% and 2 % TiO _2_NP respectively. ^45^

## Discussion

The effects of addition of different sizes (less than and greater than 50 nm) and percentages of TiO _2_NP on the antimicrobial, SH and SR of heat cured denture base resins was narratively synthesized. It was found that size of the TiO _2_NP had less influence on these properties, but with increase in the percentage of TiO _2_NP these properties were affected.

### Sample dimensions, acrylic used and treatment of NPs

Differences in the sample dimensions between the studies were observed, indicating methodological inconsistencies. Four studies mentioned the sample fabrications as per the recommended standardizations. ^47^^,^^50^^,^^52^^,^^55^ Even though the intent of the study was to include the effects of the addition of TiO _2_NP into CAD/CAM dentures, evidence on the comparison of these effects with conventional heat cured resins was found to be lacking. ^37^^,^^42^ Only one study used high impact acrylic resin and found that the hardness further increased by addition of 5 % TiO _2_NP, the difference being statistically significant. ^48^ Despite the fact that polishing of the acrylic affects the surface properties and subsequent microbial adhesion, only seven studies were found to have mentioned the procedures adopted for finishing and polishing ^44^^,^^45^^,^^49^^,^^51^^,^^53^^,^^58^ and six studies mentioned details of sample storage. ^45^^,^^46^^,^^48^^,^^52^^,^^54^

The methods of dispersion and surface treatments of NPs play a significant role in affecting the mechanical properties. NPs when treated with low molecular weight organosilicon surface treatment agents or silane coupling agents facilitate formation of a molecular bridge between the interface of the organic and inorganic substance. This results in increasing the NPs dispersion and enhanced the properties. ^60^^–^^62^ Even though the treatment can affect the resultant properties, only four studies mentioned the process of treating the NPs using silanization or any other form of surface treatment. ^44^^,^^46^^,^^47^^,^^51^ As surface treatment of the NPs has shown to improve properties, future studies must mention methods used for surface treatments.

Thorough mixing of NPs is essential as TiO _2_NP have high surface energies and agglomerate formation can act as stress concentrating centres thereby adversely affecting the properties. ^52^^,^^57^ Different methods of mixing and dispersion of the particles were used in the selected studies such as speed mixer, vacuum mixer, twin screw extruder, ultrasonication, planetary ball mill and ultrasonic homogeniser.

### Methods for testing properties

Considerable heterogeneity in methods of testing was observed in which researchers used methods such as antibacterial adhesion test, minimum inhibitory concentrations (MIC) of the NPs using microbroth dilution method and biofilm formation, MIC by broth culture, the plate count method were used. ^45^^,^^47^^,^^51^^,^^53^^,^^55^^,^^57^^,^^58^ Similarly, variation in the methods for testing SH was observed such as in Shore D hardness, ^46^^,^^50^ Vicker’s Hardness, ^48^^,^^49^^,^^54^ and Rockwell Hardness. ^56^ While, SR was evaluated using a profilometer, ^46^^,^^50^^,^^52^ perthometer, ^58^ and digital microscope. ^51^

### Effect of the size of the TiO _2_NP

The results of the current study indicate that the size of the added TiO _2_NP did not affect the observed antimicrobial effect and SH as studies that used smaller TiO _2_NP (less than 50 nm) ^52^^,^^53^^,^^55^^,^^57^^,^^58^ as well as those that used relatively larger sized NPs (greater than 50 nm), ^45^^,^^51^ reported improved antimicrobial properties. Previous studies have found that smaller sized NPs improve properties as they can penetrate between linear chains and form homogenous mixture, and additionally can easily attach and penetrate cell membranes. ^63^^–^^65^ Although studies reported conflicting results regarding the effect on SR, SH was found to increase irrespective of the size of TiO _2_NP added to PMMA. In-order to provide stronger evidence on the optimum size of NPs to achieve improved properties, future studies must justify selection of a size as well as analyse the effect of increasing or decreasing the size of NPs on the given properties.

### Effect of percentage of the addition of TiO _2_NP

Previous studies have found that a smaller percentage of the added NPs ensures that the NPs are well embedded into the resin. ^66^^,^^67^ From the analysis of the obtained results it was found that increasing the percentage of addition of TiO _2_NP did not always improve the antimicrobial effect. The most promising results were obtained with 3% of addition of TiO _2_NP, as four studies demonstrated improved antimicrobial effect with 3% TiO _2_NP. ^45^^,^^47^^,^^51^^,^^55^ The observed antimicrobial effects can be attributed to the different mechanisms of action such as the ability of TiO _2_NP to produce cytotoxic oxygen radicals, reduction in the bacterial attachment and biofilm formation onto polymer surfaces and deactivation of cellular enzymes causing to cell death. ^47^^,^^68^^–^^72^

Regarding the effect on SH, the current analysis found that although with an increase in the percentage of TiO _2_NP, the SH of the nanocomposite increases, little or no improvement occurred with percentages below 3%. The variations in the SH are said to be due to the dispersion of the NPs other factors such as use of silane coupling agent, mixing methods used for dispersion for minimum agglomerates formation and the greater presence of cross-linking in the surface layers than the internal layers of the material. ^45^^,^^73^^–^^75^

With regards to the SR, the conclusive effect of increasing the percentage could not be obtained, however, at 3% the SR was found to increase. Factors such as the dispersion of the NP to close the gap in the resin matrix, presence of the amount of nanoparticle on the surface and hydrophobicity of the nano-resin composite play important roles to determine the SR. ^76^^–^^78^ The clinical significance of this property cannot be underestimated as with the increase in the SR larger number of sites are available for microbial adhesion, however only two studies evaluated the microbial adhesion on addition of different concentrations of TiO _2_NP. ^18^^,^^51^^,^^58^^,^^79^

The current review differs from previous reviews as the effect of nanohybrid combination using TiO _2_NP was also evaluated, wherein it was observed that the nanohybrid combination of TiO _2_ and SiO _2_ showed antimicrobial action against Streptococcus M. ^57^ Studies have found that TiO _2_NP on doping with other metals or metal oxides, showed improved photocatalytic activity thereby showing improved antimicrobial properties. ^80^^,^^81^ Keeping in view with the limited available evidence but with promising results, future research on analysing combination of metal oxide NPs with TiO _2_NP can be undertaken.

### Recommendations for future clinical practice and research

Based on the results of the current systematic review, the use of TiO _2_NP size up to 100 nm and at 3% concentration can be recommended to achieve optimum antimicrobial effect, SH and SR, however, additional efforts must be undertaken to improve the SR so as to minimize the microbial adhesion. Future studies can be undertaken to assess the impact to different methods to improve the SR with their impact on the microbial adhesion.

### Strengths and Limitations

The current study has certain limitations. Firstly, due to the significant diversity within the primary studies, meta-analysis of the results could not be performed and the conclusions are drawn based on narrative synthesis. Secondly, the results of the current review must be interpreted with careful understanding as included studies showed moderate results in methodological and risk assessments due to the in-vitro nature of the majority of the studies, where uniform guidelines are currently missing. ^82^^,^^83^The strength of the current study is the use robust methodology and use of a comprehensive methodology of a literature search based on the recently introduced PRISMA-S guidelines, minimizing the publication bias and following a robust methodology in the conduction of the systematic review and provides updated information on the use of the pure form and hybrid TiO _2_NP.

## Conclusions

Based on the evidence synthesized, it can be concluded that on addition of TiO _2_NP to heat polymerized PMMA denture base resin, the antimicrobial property and SH improved irrespective of the size of the TiO _2_NP, however, addition of TiO _2_NP less than 50 nm increased the SR. Increasing the percentage of TiO _2_NP showed an increase in the SH, but did not always increase the antimicrobial property. Addition of 3% TiO _2_NP provided optimum results with regards to antimicrobial effect and SH, however, with 3% TiO _2_NP, SR was found to increase. Due to insufficient data currently available, the effect of addition of TiO _2_NP on CAD/CAM PMMA denture base resins could not be established.

## Data Availability

No underlying data are associated with this article. OSF: Effect of addition of titanium dioxide nanoparticles on the antimicrobial properties, surface roughness and surface hardness of polymethyl methacrylate: A systematic Review. DOI: 10.17605/OSF.IO/AV56D (Pragati, 2022). This project contains the following underlying data:
• PRISMA Flowchart 2020 (Flowchart of all the sources searched and the results obtained) • PRISMA 2020 Checklist (Checklist of reporting guidelines) •
Table 1 and 2 (Data Extraction Sheet of the effect of titanium dioxide nanoparticles on the antimicrobial properties, surface hardness and surface roughness) • Supplementary Material (Details of search strategies used, peer review of the search strategy, list of included and studies) Data are available under the terms of the Creative Commons Zero “No rights reserved” data waiver (CC0 1.0 Public domain dedication).
