# Supplementary material for: Effect of addition of titanium dioxide nanoparticles on the antimicrobial properties, surface roughness and surface hardness of polymethyl methacrylate: A Systematic Review
Source: F1000Res. 2023 May 31;12:577. [Version 1] doi: 10.12688/f1000research.130028.1 (PMC10323281; doi:10.12688/f1000research.130028.1)
Supplement: Peer review history

# Peer review: Reviewer response for version 1

**DOI:** 10.5256/f1000research.142755.r189294

**Published:** 2023-08-08

**Authors:** Carlos Enrique Cuevas Suárez

**Affiliations:** 1 Autonomous University of Hidalgo State. Circuito Ex Hacienda La Concepción, San Agustín Tlaxiaca, Mexico

Please consider to resolve the following issues:

1. Please consider to state a null hypothesis for the study.
2. Authors stay "Studies based on TiO _2_ nanotubes, fillers, **fibres** and coating on PMMA were excluded.". However, for control, it's stated that heat polymerized PMMA without any addition of NPs or **fibre**, were included. So, it's difficult to understand if the works using TiO2 fibres were included.
3. Authors stay as control the use of heat polymerized PMMA. Does the cold-cure PMMA was excluded?
4. In the PICO strategy, the intervention should be:  Addition of TiO _2_NP in pure or hybrid form in heat-polymerized PMMA.
5. The risk of bias analysis should be performed again using specific tools. My suggestion is to use the RoNDEMAT tool for the analysis of the in-vitro studies, while the clinical trial should be assessed using the RoB 2 tool.
6. Please include a Table summarizing the main characteristics of the studies. 
7. Some parts of the discussion should be moved to the results section; i.e. Sample dimensions, acrylic used and treatment of NPs; Methods for testing properties.
8. The conclusion section should be re-written again. Without a proper statistical analysis, the authors are not able to determine which concentration of NP's are optimal for improving the properties of the material.
9. I suggest that the entire manuscript be proof-read. Some parts of the manuscript are difficult to read. 

Are the rationale for, and objectives of, the Systematic Review clearly stated?

Yes

Is the statistical analysis and its interpretation appropriate?

Not applicable

Are sufficient details of the methods and analysis provided to allow replication by others?

Yes

Are the conclusions drawn adequately supported by the results presented in the review?

Partly

Reviewer Expertise:

Adhesive dentistry

I confirm that I have read this submission and believe that I have an appropriate level of expertise to confirm that it is of an acceptable scientific standard, however I have significant reservations, as outlined above.

# Peer review: Reviewer response for version 1

**DOI:** 10.5256/f1000research.142755.r179872

**Published:** 2023-07-05

**Authors:** Rekha Gupta, Modhupa Ghosh

**Affiliations:** 1 Department of Prosthodontics, Maulana Azad Institute of Dental Sciences, New Delhi, Delhi, India; 2 Prosthodontics, Maulana Azad Institute of Dental Sciences, New Delhi, Delhi, India

The systematic review has been presented in a detailed and precise manner and deals with an important aspect of denture fabrication and its subsequent use. The inclusion and exclusion criteria along with the search strategy have been sufficiently described and are reproducible. The prescribed guidelines have also been followed.

 Minor modifications are suggested as under:

Results:

 "With regards to the SR, Cascione and others found a decrease in SR with large sized TiO2NP (greater or equal to 50 nm), but three studies reported an increase in SR with the addition of less than 50 nm TiO2NPand one study found in-significant improvement in SR.”

 The statement, though seemingly states that a decrease in particle size has been found to be associated with an increase in surface roughness (SR), has been written in a contradictory and confusing way. The authors may modify the language for better understanding.

 In, Recommendations for future clinical practice and research:

 “Based on the results of the current systematic review, the use of TiO2NP size up to 100 nm and at 3% concentration can be recommended to achieve optimum antimicrobial effect, SH and SR, however, additional efforts must be undertaken to improve the SR so as to minimize the microbial adhesion.”

 However, in Conclusion the authors state that:

 “Based on the evidence synthesized, it can be concluded that on addition of TiO2NP to heat polymerized PMMA denture base resin, the antimicrobial property and SH improved irrespective of the size of the TiO2NP, however, addition of TiO2NP less than 50 nm increased the SR.”

 The authors need to clarify the proposed size of the nanoparticles based on the findings of Surface roughness as the results vary at <50nm and >50nm particle size, with the antimicrobial property and SH remaining unaffected.

Are the rationale for, and objectives of, the Systematic Review clearly stated?

Yes

Is the statistical analysis and its interpretation appropriate?

Not applicable

Are sufficient details of the methods and analysis provided to allow replication by others?

Yes

Are the conclusions drawn adequately supported by the results presented in the review?

Yes

Reviewer Expertise:

Clinical research related to oral health problems, maxillofacial Prosthodontics, Dental Implants, Dental materials used in Prosthodontics

We confirm that we have read this submission and believe that we have an appropriate level of expertise to confirm that it is of an acceptable scientific standard.

# Peer review: Reviewer response for version 1

**DOI:** 10.5256/f1000research.142755.r179884

**Published:** 2023-06-30

**Authors:** Veena B. Benakatti

**Affiliations:** 1 Department of Prosthodontics and Crown and Bridge, KAHER'S KLE VK Institute of Dental Sciences, Belagavi, Karnataka, India

It is a well-conducted research with a robust methodology. The findings of the study would benefit the field of dental science.

 The methodology and findings/values of the antimicrobial property, surface hardness, and surface roughness of the individual studies can be summarized in a table that will give a general view of the selected studies.

Are the rationale for, and objectives of, the Systematic Review clearly stated?

Yes

Is the statistical analysis and its interpretation appropriate?

Yes

Are sufficient details of the methods and analysis provided to allow replication by others?

Yes

Are the conclusions drawn adequately supported by the results presented in the review?

Yes

Reviewer Expertise:

Artificial intelligence in Dentistry

I confirm that I have read this submission and believe that I have an appropriate level of expertise to confirm that it is of an acceptable scientific standard.
